# Hospital-Acquired COVID-19 Infection Increases Morbidity and Mortality: A Case Report of Post-Surgical Challenge of Duodenal Ulcer Repair During COVID-19 Era

**DOI:** 10.7759/cureus.22646

**Published:** 2022-02-27

**Authors:** Jamil F Ghanayem, Andee Dzulkarnaen Zakaria, Wan Zainira Wan Zain, Muath Mamdouh Mahmod Al-Chalabi

**Affiliations:** 1 General Surgery, Universiti Sains Malaysia (USM), Kota Bharu, MYS; 2 Colorectal Surgery, Hospital Universiti Sains Malaysia, Kota Bharu, MYS; 3 Reconstructive Sciences Unit, Universiti Sains Malaysia (USM), Kota Bharu, MYS

**Keywords:** hospital-acquired covid-19 infection, covid-19 pandemic, surgical acute abdomen, acute abdomen in covid-19, perforated duodenal ulcer

## Abstract

Hospital-acquired infections are nosocomially acquired infections that are not present or incubating at the time of admission to a hospital. During the COVID-19 pandemic, many hospitals became sources of the infection, creating a great challenge for health care providers and uninfected patients who visited these hospitals seeking medical or surgical advice. We are presenting a middle-aged man who complained of abdominal pain associated with poor oral intake during the COVID-19 pandemic in January 2021. After being diagnosed with a perforated duodenal ulcer, he underwent laparoscopic repair. He was postoperatively referred to interventional radiology for central line insertion. However, as one of the pre-procedure perquisites during the COVID-19 pandemic, he underwent a nasopharyngeal swab real-time PCR test, which was positive for COVID-19 infection to be considered hospital-acquired. This article shows how the pandemic may complicate the post-surgical condition, increasing patient morbidity and mortality.

## Introduction

Coronavirus disease 2019 (COVID-19), the highly contagious viral illness caused by severe acute respiratory syndrome coronavirus 2 (SARS-CoV-2) started in December 2019 and was declared a global pandemic in March 2020 by WHO. It led to catastrophic effects and resulted in millions of deaths worldwide, emerging as the most consequential global health crisis since the era of the influenza pandemic of 1918 [1]. There are potential morbidity and mortality risks for post-operative patients who acquire COVID-19 infection nosocomially, leading to rapidly deteriorating post-operative complications. Herein, we share our experience managing a patient with a perforated duodenal ulcer who underwent laparoscopic surgical repair for his perforated duodenal ulcer during the COVID-19 pandemic. For post-operative follow-up purposes, the patient was sent to the interventional radiology department for a peripherally inserted central catheter, which required a nasopharyngeal swab for real-time PCR (RT-PCR) to follow pandemic pre-procedural protocol. The result was positive for COVID-19 infection. This condition alerts healthcare providers to take the necessary precautions to avoid the hospital-acquired COVID-19 infection [2]. In this case, the acquired COVID-19 infection and ICU admission worsened the patient's lung condition. Conclusively, the COVID-19 infection was the leading cause of patient mortality.

## Case presentation

A 46-year-old Indian male presented to the emergency department complaining of right hypochondrial pain for three days. The pain was colicky, with a score of 8/10, radiating to the back and tip of the shoulder, relieved upon forward-leaning, aggravated after taking a meal, associated with nausea, and reduced oral intake for three days. Upon arrival, GCS was full, BP 110/68 mmHg, HR 92 bpm, Hb 11.1 g/dL. A chest X-ray showed a clear lung field with no air under the diaphragm. Bedside ultrasound showed the presence of free intraperitoneal fluid in Morison's pouch. The initial impression at the emergency department was of an acute abdomen, which needed further workup to rule out perforated viscus. The case was sent to a surgical team, and the first impression was that the patient had acute cholecystitis.

Ultrasonography of the hepatobiliary system showed a gall bladder polyp with no evidence of cholecystitis, cholelithiasis, or choledocholithiasis. Other things that were found were a thickened pyloric wall with no apparent inside mass, complex ascites, and simple renal cysts on both sides.

**Figure 1:**
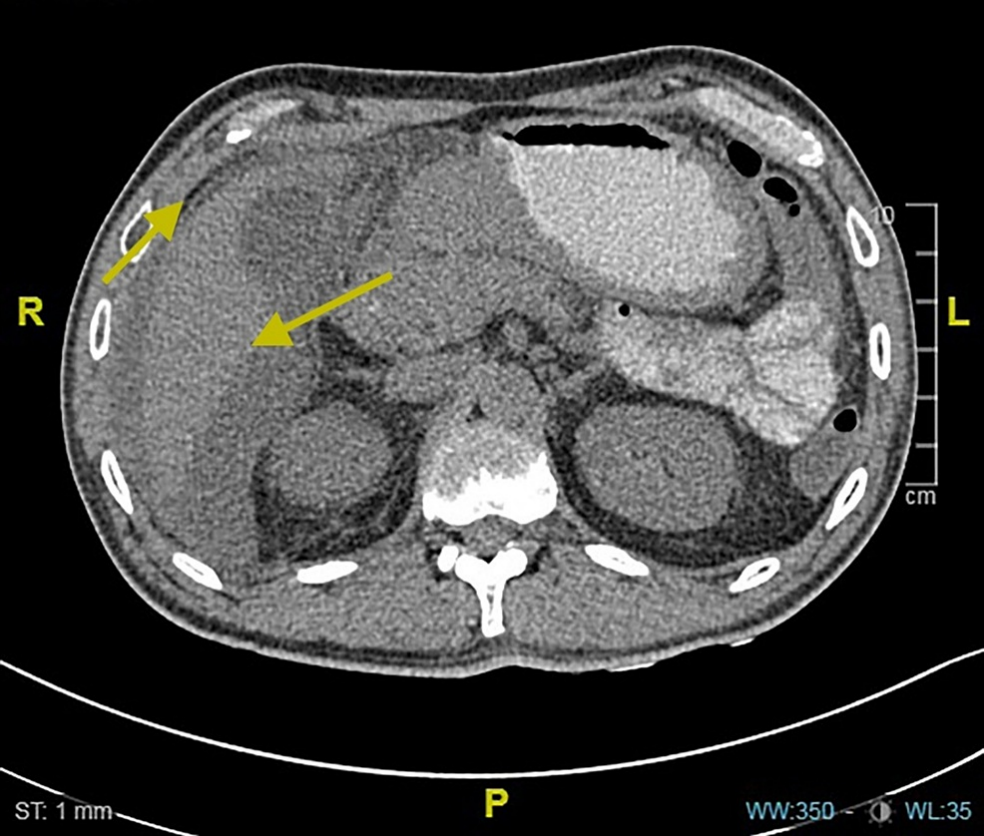
Abdominal CT-scan shows pneumoperitoneum secondary to perforated viscus (right yellow arrow) and peri-hepatic free fluid with sub-capsular liver collection (left yellow arrow).

Computed tomography of the abdomen (Figure 1) showed pneumoperitoneum likely secondary to perforated viscus, mainly from the anterior segment of the duodenal wall, and peri-hepatic free fluid with the sub-capsular liver collection.

**Figure 2:**
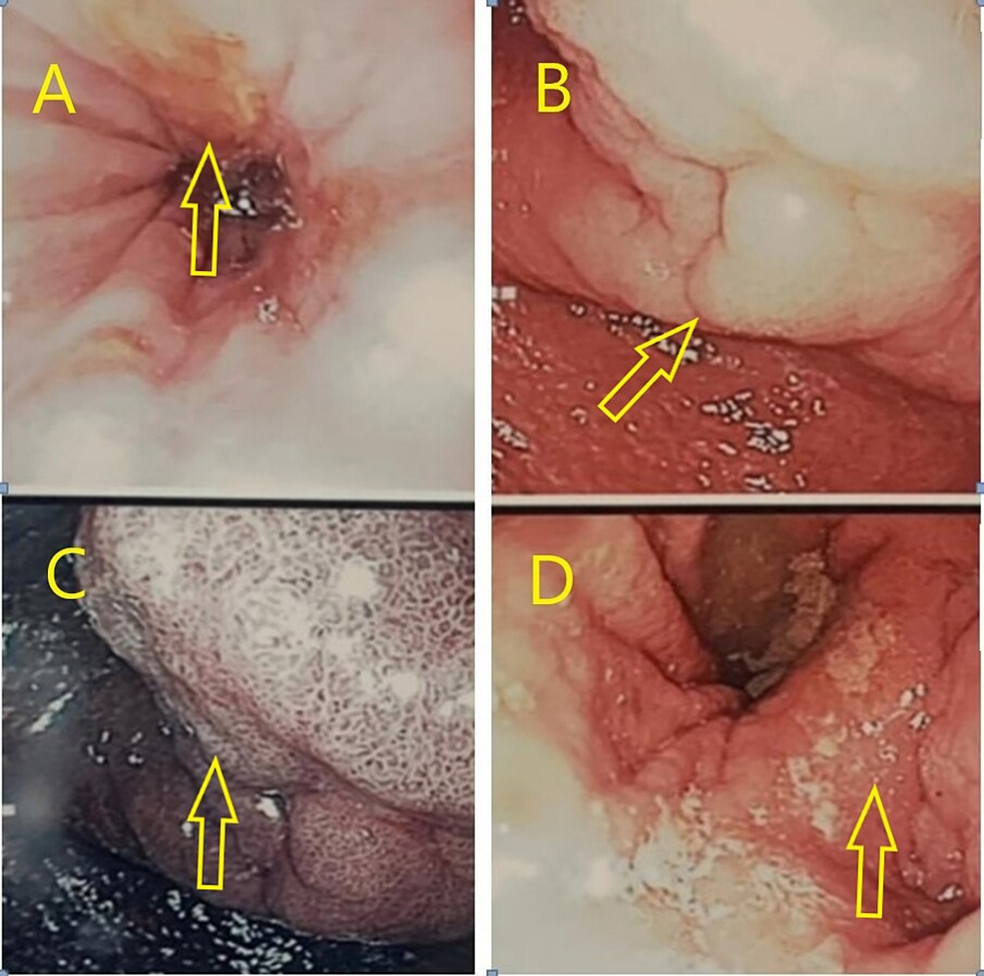
Esophagogastroduodenoscopy shows (A) multiple small ulcers with erosions at the gastroesophageal junction, (B) polypoidal growth at the pylorus, (C) tattooed appearance of the polypoidal growth of pylorus, and (D) distal pylorus with multiple erosions.

The patient underwent laparoscopic perforated duodenal ulcer repair. The intraoperative findings included duodenal perforation measuring 0.5 × 0.5 cm^2^ repaired using V-lock 2/0 wound closure, pre-pyloric region thickening, localized contamination with pus and slough at sub-phrenic and sub-hepatic regions, and adhered omentum to the perforation site and gall bladder with dense adhesion between the omentum and anterior abdominal wall over the previous appendicectomy site. On day 3 postoperatively, the patient was unable to start oral intake. Therefore, an upper gastrointestinal contrast study was performed and showed that the contrast pooled in the stomach and did not pass through the pylorus. Total parenteral nutrition started because of gastric outlet obstruction. On day 5 postoperatively, esophagogastroduodenoscopy was performed (Figure 2), and the findings were polypoidal growth at the pylorus with distorted antrum and pylorus of the stomach and a few small ulcers at the gastroesophageal junction.

**Figure 3:**
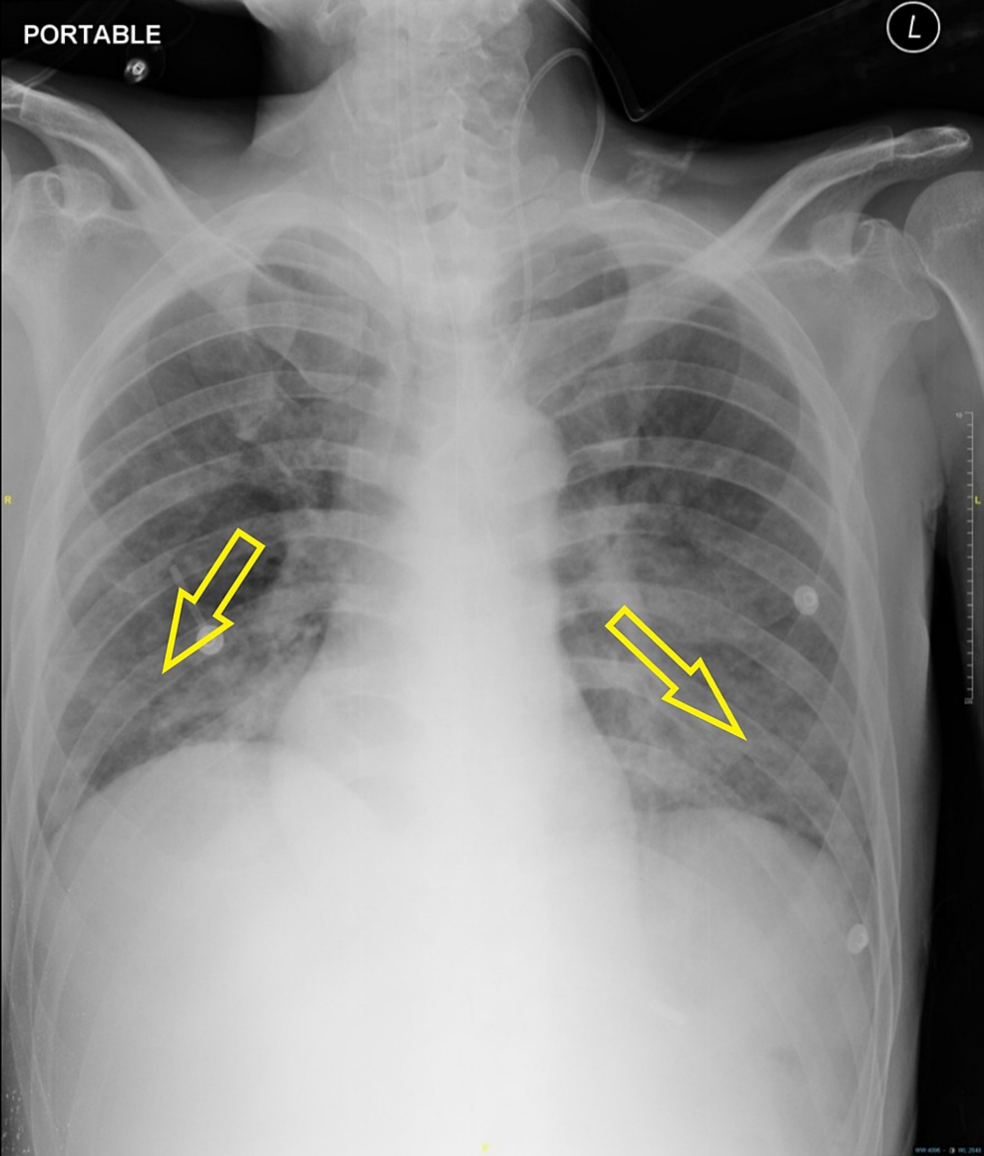
Chest X-ray shows bilateral lower zone ground-glass opacities (yellow arrows).

The patient was scheduled for a peripherally inserted central catheter under interventional radiology, but because of the COVID-19 pandemic, the protocol was to perform a pre-procedural COVID-19 RTK Ag test. Unfortunately, the result was a positive COVID-19 infection. The patient was transferred to the isolation ward and subsequently suffered from episodes of oxygen desaturation. Chest X-rays showed bilateral lower zone ground-glass opacities (Figure 3).

Immediately, the antiviral Favipiravir 800 mg and the antibacterial Piperacillin/Tazobactam 4.5 g started. Blood culture and sensitivity showed no growth. C-reactive protein (CRP) was 12 mg/L. However, the patient developed septicemic shock secondary to hospital-acquired COVID-19 infection.

The condition rapidly deteriorated as the patient developed infective diarrhea, concomitant with hospital-acquired COVID-19 infection. Computed tomography of the thorax showed multiple bilateral lung nodules with consolidation, suggestive of infective changes. The patient was intubated due to respiratory distress post-hospital-acquired COVID-19 infection. A few days later, a chest X-ray showed worsening cavitation in the right upper lobe of the lung, suggestive of necrotizing pneumonia. However, supported treatment continued with inotropes, but the patient's vitals unfortunately continued to worsen until he passed away. The patient's condition deteriorates in tandem as the COVID-19 infection impairs lung function, causes septic shock, multi-organ failure, and eventually death.

## Discussion

The surgical disciplines face significant challenges from the COVID-19 pandemic, and the effects on the surgical profession will be lasting. The long-term impact on patients with surgical disease has yet to be fully realized. However, operating on patients with COVID-19 is linked with considerably increased odds of morbidity and mortality [3].

As the COVID-19 pandemic remains and surges, we need to balance patients’ surgical needs with COVID-19-specific risks in the context of a strained health care system, remembering that COVID-19 infection is an independent risk factor for surgical mortality [4]. From the pandemic of COVID-19, it was clear that hospitals were an essential factor for viral spread to patients with prolonged stays and underlying chronic diseases. However, overall hospital transmission of SARS-CoV-2 in personal protective equipment is likely rare, even during periods of high community spreading.

Operating on asymptomatic or symptomatic COVID-19 patients raises the risk of perioperative morbidity and mortality. The common serious complication of COVID-19 infection is pneumonia, presenting primarily with fatigue, dry cough, and dyspnea and ending with severe respiratory damage [3].

Still, the data regarding surgical patients who acquired COVID-19 from the hospital are limited. Doglietto et al. [5] mentioned that few recent literature reports discuss postoperative complications with COVID-19 infection. In Iran at the beginning of the COVID-19 outbreak, two of three patients died due to postoperative fever and pulmonary complications after uneventful elective general surgery. While in Wuhan, 34 patients who underwent elective surgery during the incubation period of COVID-19 in four hospitals developed pneumonia shortly after surgery; 15 (44.1%) required admission to the ICU; and 7 (20.5%) ultimately died. Another report mentioned that the mortality rate among 13 patients who underwent elective thoracic surgery was 38.5%.

This high mortality rate in surgical patients with COVID-19 should warn the surgical care provider to inform the patient of their higher in-hospital mortality risk [6]. More importantly, postponing surgery should be recommended for patients with a positive preoperative COVID-19 test result when possible, unless surgical intervention is necessary for life-saving measures. Another big challenge for surgery has been how to stop non-urgent and non-emergency surgery safely.

## Conclusions

Once a surgical patient with an emergency condition is admitted, all parameters and precautions should be taken to avoid transmission of COVID-19 infection because acquiring COVID-19 infection carries potential risks and post-operative complications. Surgical health providers should be aware that the transmission of the hospital-acquired COVID-19 infection is possible and expected during the COVID-19 pandemic, especially in critically ill surgical patients. In addition, the control system on the visitors should be restricted to avoid outside transmission of the COVID-19 infection.
